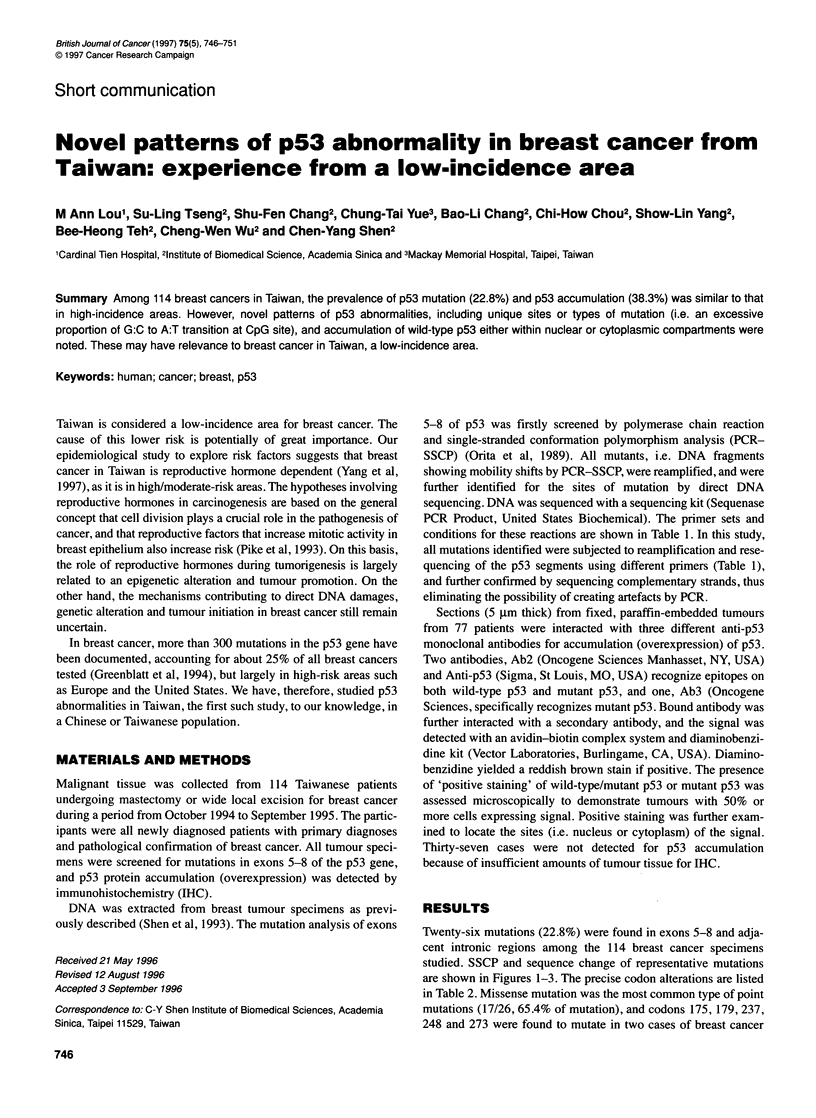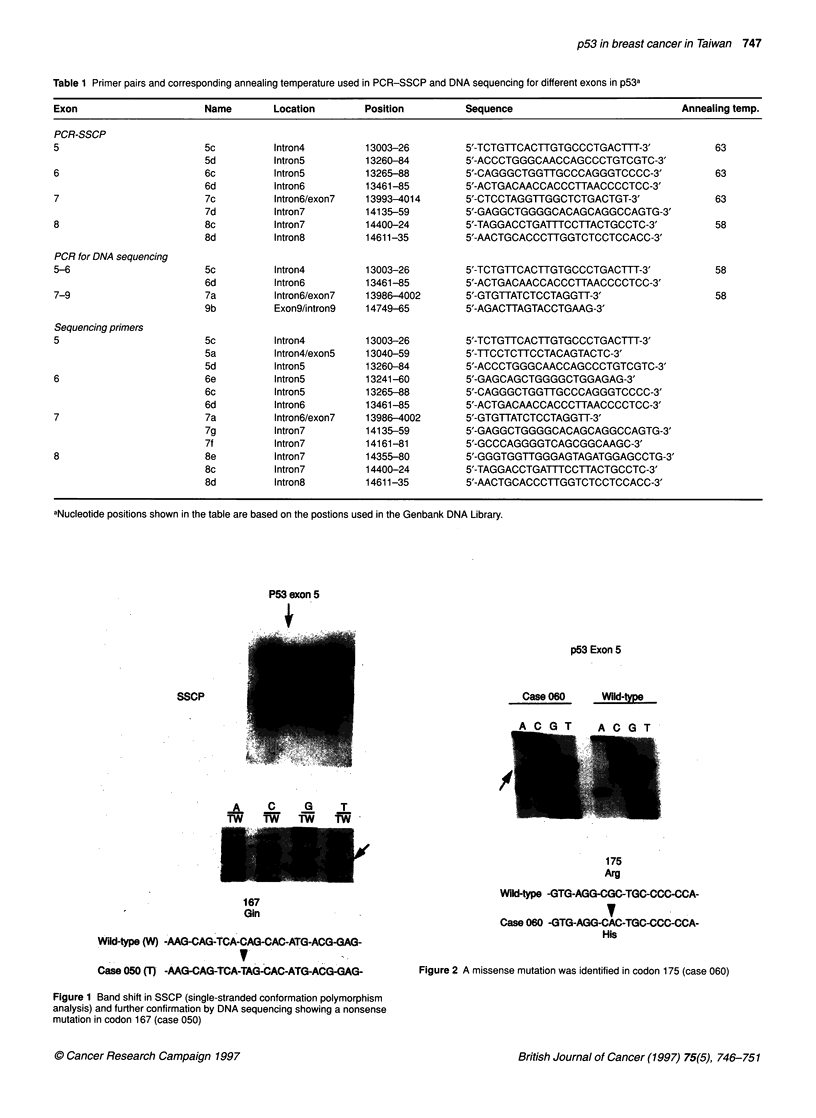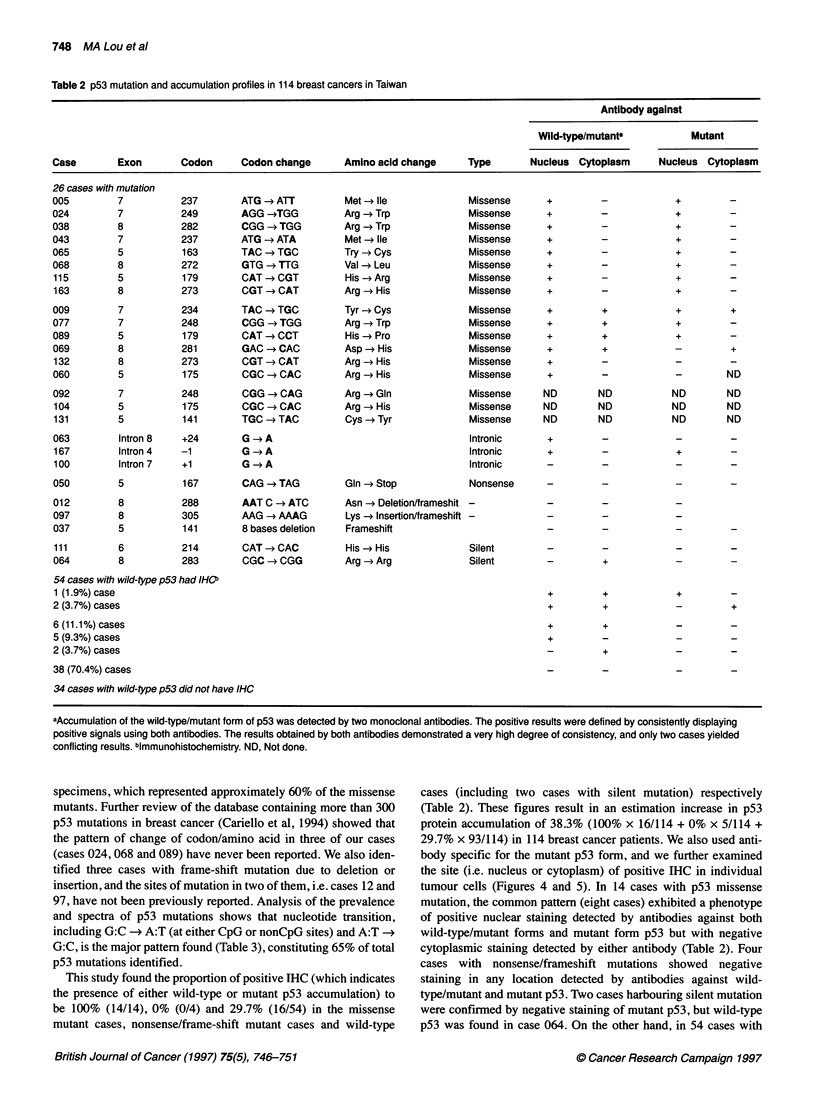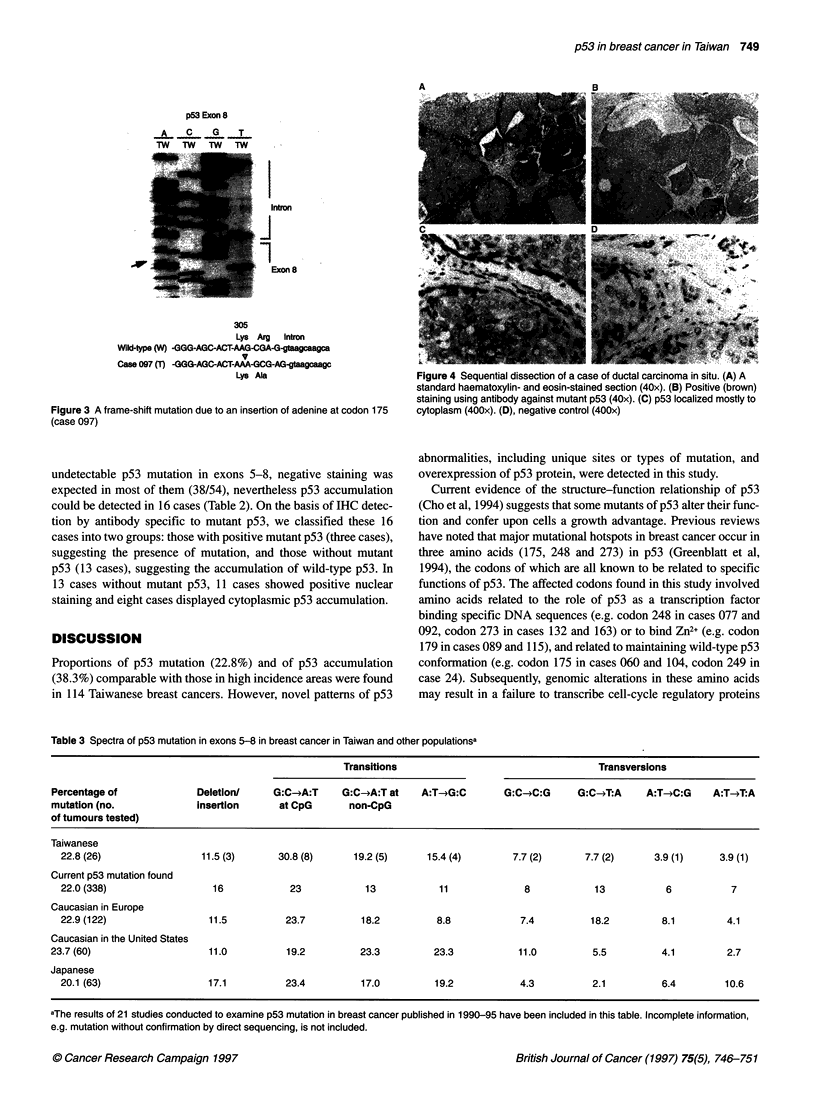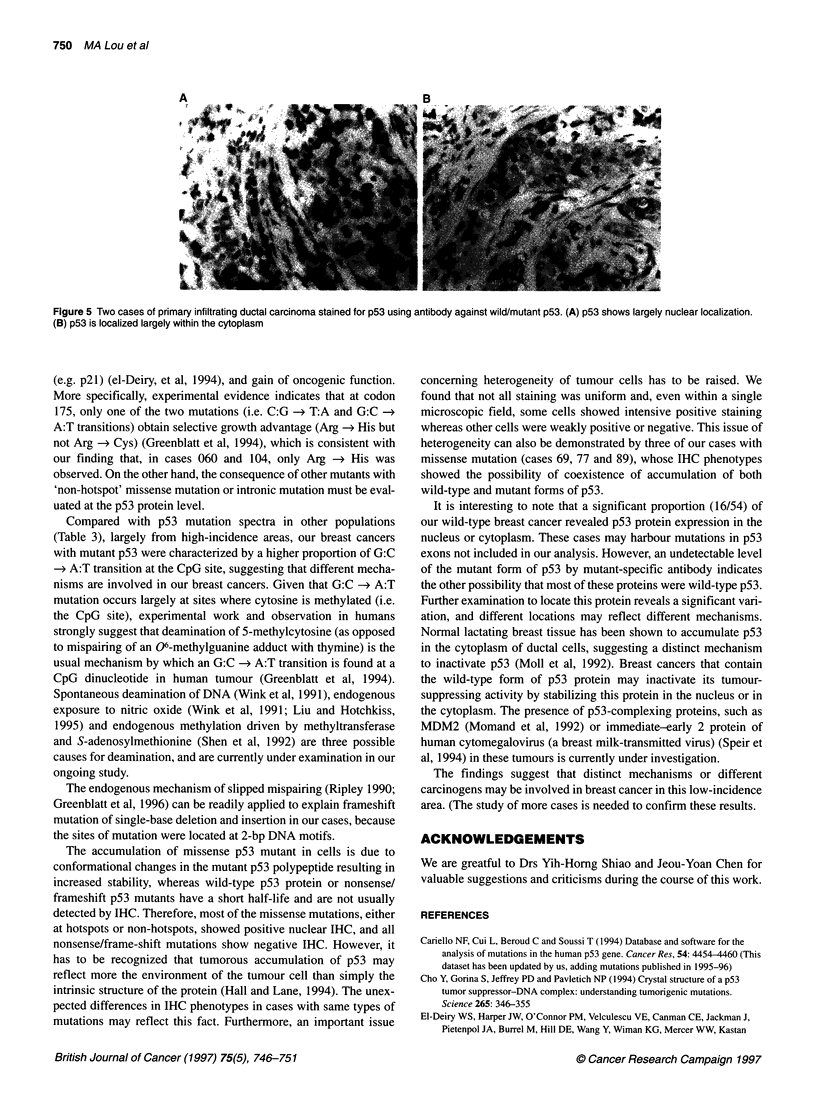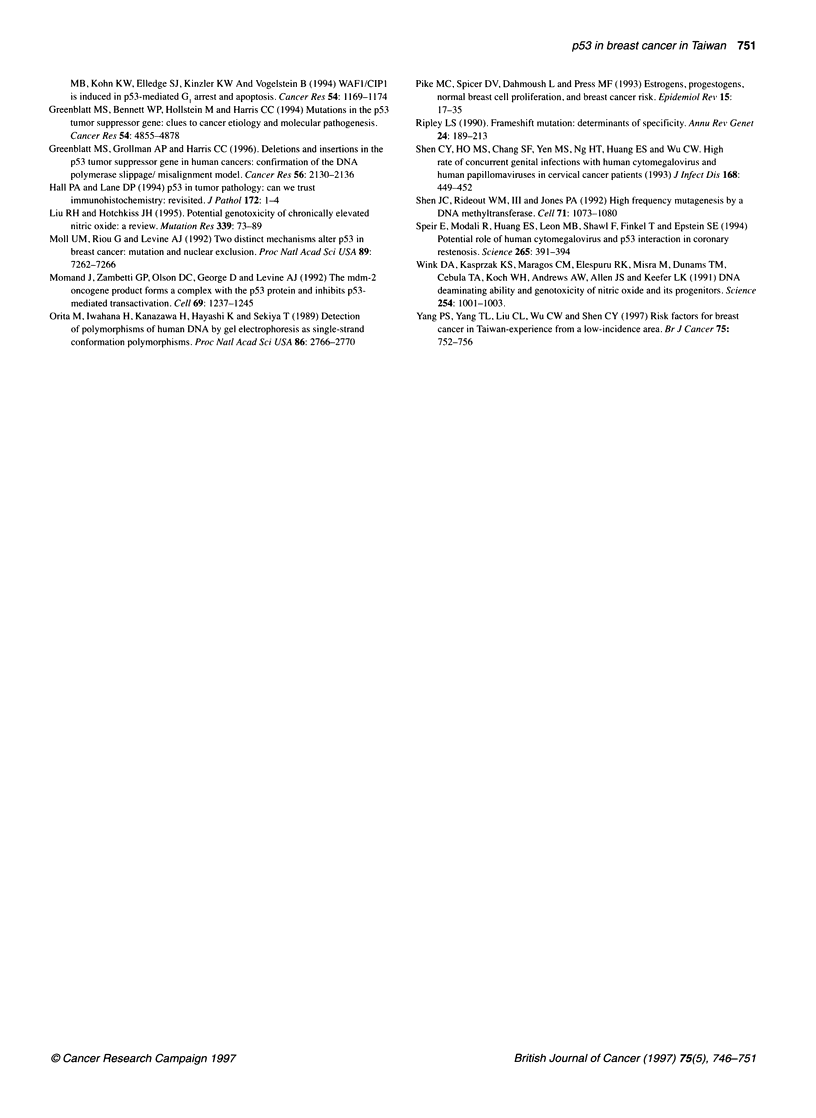# Novel patterns of p53 abnormality in breast cancer from Taiwan: experience from a low-incidence area.

**DOI:** 10.1038/bjc.1997.132

**Published:** 1997

**Authors:** M. A. Lou, S. L. Tseng, S. F. Chang, C. T. Yue, B. L. Chang, C. H. Chou, S. L. Yang, B. H. Teh, C. W. Wu, C. Y. Shen

**Affiliations:** Cardinal Tien Hospital, Taipei, Taiwan.

## Abstract

**Images:**


British Journal of Cancer (1997) 75(5), 746–751
© 1997 Cancer Research Campaign

Short communication

### Novel patterns of p53 abnormality in breast cancer from Taiwan: experience from a low-incidence area

M Ann Lou[1], Su-Ling Tseng[2], Shu-Fen Chang[2], Chung-Tai Yue[3], Bao-Li Chang[2], Chi-How Chou[2], Show-Lin Yang[2], Bee-Heong Teh[2], Cheng-Wen Wu[2] and Chen-Yang Shen[2]

[1]Cardinal Tien Hospital, [2]Institute of Biomedical Science, Academia Sinica and [3]Mackay Memorial Hospital, Taipei, Taiwan

**Summary** Among 114 breast cancers in Taiwan, the prevalence of p53 mutation (22.8%) and p53 accumulation (38.3%) was similar to that in high-incidence areas. However, novel patterns of p53 abnormalities, including unique sites or types of mutation (i.e. an excessive proportion of G:C to A:T transition at CpG site), and accumulation of wild-type p53 either within nuclear or cytoplasmic compartments were noted. These may have relevance to breast cancer in Taiwan, a low-incidence area.

**Keywords:** human; cancer; breast, p53

Taiwan is considered a low-incidence area for breast cancer. The cause of this lower risk is potentially of great importance. Our epidemiological study to explore risk factors suggests that breast cancer in Taiwan is reproductive hormone dependent (Yang et al, 1997), as it is in high/moderate-risk areas. The hypotheses involving reproductive hormones in carcinogenesis are based on the general concept that cell division plays a crucial role in the pathogenesis of cancer, and that reproductive factors that increase mitotic activity in breast epithelium also increase risk (Pike et al, 1993). On this basis, the role of reproductive hormones during tumorigenesis is largely related to an epigenetic alteration and tumour promotion. On the other hand, the mechanisms contributing to direct DNA damages, genetic alteration and tumour initiation in breast cancer still remain uncertain.

In breast cancer, more than 300 mutations in the p53 gene have been documented, accounting for about 25% of all breast cancers tested (Greenblatt et al, 1994), but largely in high-risk areas such as Europe and the United States. We have, therefore, studied p53 abnormalities in Taiwan, the first such study, to our knowledge, in a Chinese or Taiwanese population.

### MATERIALS AND METHODS

Malignant tissue was collected from 114 Taiwanese patients undergoing mastectomy or wide local excision for breast cancer during a period from October 1994 to September 1995. The participants were all newly diagnosed patients with primary diagnoses and pathological confirmation of breast cancer. All tumour specimens were screened for mutations in exons 5–8 of the p53 gene, and p53 protein accumulation (overexpression) was detected by immunohistochemistry (IHC).

DNA was extracted from breast tumour specimens as previously described (Shen et al, 1993). The mutation analysis of exons 5–8 of p53 was firstly screened by polymerase chain reaction and single-stranded conformation polymorphism analysis (PCR–SSCP) (Orita et al, 1989). All mutants, i.e. DNA fragments showing mobility shifts by PCR–SSCP, were reamplified, and were further identified for the sites of mutation by direct DNA sequencing. DNA was sequenced with a sequencing kit (Sequenase PCR Product, United States Biochemical). The primer sets and conditions for these reactions are shown in Table 1. In this study, all mutations identified were subjected to reamplification and resequencing of the p53 segments using different primers (Table 1), and further confirmed by sequencing complementary strands, thus eliminating the possibility of creating artefacts by PCR.

Sections (5 μm thick) from fixed, paraffin-embedded tumours from 77 patients were interacted with three different anti-p53 monoclonal antibodies for accumulation (overexpression) of p53. Two antibodies, Ab2 (Oncogene Sciences Manhasset, NY, USA) and Anti-p53 (Sigma, St Louis, MO, USA) recognize epitopes on both wild-type p53 and mutant p53, and one, Ab3 (Oncogene Sciences, specifically recognizes mutant p53. Bound antibody was further interacted with a secondary antibody, and the signal was detected with an avidin–biotin complex system and diaminobenzidine kit (Vector Laboratories, Burlingame, CA, USA). Diaminobenzidine yielded a reddish brown stain if positive. The presence of 'positive staining' of wild-type/mutant p53 or mutant p53 was assessed microscopically to demonstrate tumours with 50% or more cells expressing signal. Positive staining was further examined to locate the sites (i.e. nucleus or cytoplasm) of the signal. Thirty-seven cases were not detected for p53 accumulation because of insufficient amounts of tumour tissue for IHC.

### RESULTS

Twenty-six mutations (22.8%) were found in exons 5–8 and adjacent intronic regions among the 114 breast cancer specimens studied. SSCP and sequence change of representative mutations are shown in Figures 1–3. The precise codon alterations are listed in Table 2. Missense mutation was the most common type of point mutations (17/26, 65.4% of mutation), and codons 175, 179, 237, 248 and 273 were found to mutate in two cases of breast cancer

Received 21 May 1996
Revised 12 August 1996
Accepted 3 September 1996

Correspondence to: C-Y Shen Institute of Biomedical Sciences, Academia Sinica, Taipei 11529, Taiwan

**Table 1** Primer pairs and corresponding annealing temperature used in PCR–SSCP and DNA sequencing for different exons in p53[a]

| Exon | Name | Location | Position | Sequence | Annealing temp. |
|---|---|---|---|---|---|
| *PCR-SSCP* | | | | | |
| 5 | 5c | Intron4 | 13003–26 | 5′-TCTGTTCACTTGTGCCCTGACTTT-3′ | 63 |
| | 5d | Intron5 | 13260–84 | 5′-ACCCTGGGCAACCAGCCCTGTCGTC-3′ | |
| 6 | 6c | Intron5 | 13265–88 | 5′-CAGGGCTGGTTGCCCAGGGTCCCC-3′ | 63 |
| | 6d | Intron6 | 13461–85 | 5′-ACTGACAACCACCCTTAACCCCTCC-3′ | |
| 7 | 7c | Intron6/exon7 | 13993–4014 | 5′-CTCCTAGGTTGGCTCTGACTGT-3′ | 63 |
| | 7d | Intron7 | 14135–59 | 5′-GAGGCTGGGGCACAGCAGGCCAGTG-3′ | |
| 8 | 8c | Intron7 | 14400–24 | 5′-TAGGACCTGATTTCCTTACTGCCTC-3′ | 58 |
| | 8d | Intron8 | 14611–35 | 5′-AACTGCACCCTTGGTCTCCTCCACC-3′ | |
| *PCR for DNA sequencing* | | | | | |
| 5–6 | 5c | Intron4 | 13003–26 | 5′-TCTGTTCACTTGTGCCCTGACTTT-3′ | 58 |
| | 6d | Intron6 | 13461–85 | 5′-ACTGACAACCACCCTTAACCCCTCC-3′ | |
| 7–9 | 7a | Intron6/exon7 | 13986–4002 | 5′-GTGTTATCTCCTAGGTT-3′ | 58 |
| | 9b | Exon9/intron9 | 14749–65 | 5′-AGACTTAGTACCTGAAG-3′ | |
| *Sequencing primers* | | | | | |
| 5 | 5c | Intron4 | 13003–26 | 5′-TCTGTTCACTTGTGCCCTGACTTT-3′ | |
| | 5a | Intron4/exon5 | 13040–59 | 5′-TTCCTCTTCCTACAGTACTC-3′ | |
| | 5d | Intron5 | 13260–84 | 5′-ACCCTGGGCAACCAGCCCTGTCGTC-3′ | |
| 6 | 6e | Intron5 | 13241–60 | 5′-GAGCAGCTGGGGCTGGAGAG-3′ | |
| | 6c | Intron5 | 13265–88 | 5′-CAGGGCTGGTTGCCCAGGGTCCCC-3′ | |
| | 6d | Intron6 | 13461–85 | 5′-ACTGACAACCACCCTTAACCCCTCC-3′ | |
| 7 | 7a | Intron6/exon7 | 13986–4002 | 5′-GTGTTATCTCCTAGGTT-3′ | |
| | 7g | Intron7 | 14135–59 | 5′-GAGGCTGGGGCACAGCAGGCCAGTG-3′ | |
| | 7f | Intron7 | 14161–81 | 5′-GCCCAGGGGTCAGCGGCAAGC-3′ | |
| 8 | 8e | Intron7 | 14355–80 | 5′-GGGTGGTTGGGAGTAGATGGAGCCTG-3′ | |
| | 8c | Intron7 | 14400–24 | 5′-TAGGACCTGATTTCCTTACTGCCTC-3′ | |
| | 8d | Intron8 | 14611–35 | 5′-AACTGCACCCTTGGTCTCCTCCACC-3′ | |

[a]Nucleotide positions shown in the table are based on the postions used in the Genbank DNA Library.

**Figure 1** Band shift in SSCP (single-stranded conformation polymorphism analysis) and further confirmation by DNA sequencing showing a nonsense mutation in codon 167 (case 050)

**Figure 2** A missense mutation was identified in codon 175 (case 060)

© Cancer Research Campaign 1997

**Table 2** p53 mutation and accumulation profiles in 114 breast cancers in Taiwan

| | | | | | | Antibody against | | | |
|---|---|---|---|---|---|---|---|---|---|
| | | | | | | Wild-type/mutant[a] | | Mutant | |
| **Case** | **Exon** | **Codon** | **Codon change** | **Amino acid change** | **Type** | **Nucleus** | **Cytoplasm** | **Nucleus** | **Cytoplasm** |
| *26 cases with mutation* | | | | | | | | | |
| 005 | 7 | 237 | ATG → ATT | Met → Ile | Missense | + | – | + | – |
| 024 | 7 | 249 | AGG →TGG | Arg → Trp | Missense | + | – | + | – |
| 038 | 8 | 282 | CGG → TGG | Arg → Trp | Missense | + | – | + | – |
| 043 | 7 | 237 | ATG → ATA | Met → Ile | Missense | + | – | + | – |
| 065 | 5 | 163 | TAC → TGC | Try → Cys | Missense | + | – | + | – |
| 068 | 8 | 272 | GTG → TTG | Val → Leu | Missense | + | – | + | – |
| 115 | 5 | 179 | CAT → CGT | His → Arg | Missense | + | – | + | – |
| 163 | 8 | 273 | CGT → CAT | Arg → His | Missense | + | – | + | – |
| 009 | 7 | 234 | TAC → TGC | Tyr → Cys | Missense | + | + | + | + |
| 077 | 7 | 248 | CGG → TGG | Arg → Trp | Missense | + | + | + | – |
| 089 | 5 | 179 | CAT → CCT | His → Pro | Missense | + | + | + | – |
| 069 | 8 | 281 | GAC → CAC | Asp → His | Missense | + | + | – | + |
| 132 | 8 | 273 | CGT → CAT | Arg → His | Missense | + | – | – | – |
| 060 | 5 | 175 | CGC → CAC | Arg → His | Missense | + | – | – | ND |
| 092 | 7 | 248 | CGG → CAG | Arg → Gln | Missense | ND | ND | ND | ND |
| 104 | 5 | 175 | CGC → CAC | Arg → His | Missense | ND | ND | ND | ND |
| 131 | 5 | 141 | TGC → TAC | Cys → Tyr | Missense | ND | ND | ND | ND |
| 063 | Intron 8 | +24 | G → A | | Intronic | + | – | – | – |
| 167 | Intron 4 | –1 | G → A | | Intronic | + | – | + | – |
| 100 | Intron 7 | +1 | G → A | | Intronic | – | – | – | – |
| 050 | 5 | 167 | CAG → TAG | Gln → Stop | Nonsense | – | – | – | – |
| 012 | 8 | 288 | AAT C → ATC | Asn → Deletion/frameshit | – | – | – | – | |
| 097 | 8 | 305 | AAG → AAAG | Lys → Insertion/frameshift | – | – | – | – | |
| 037 | 5 | 141 | 8 bases deletion | Frameshift | | – | – | – | – |
| 111 | 6 | 214 | CAT → CAC | His → His | Silent | – | – | – | – |
| 064 | 8 | 283 | CGC → CGG | Arg → Arg | Silent | – | + | – | – |
| *54 cases with wild-type p53 had IHC*[b] | | | | | | | | | |
| 1 (1.9%) case | | | | | | + | + | + | – |
| 2 (3.7%) cases | | | | | | + | + | – | + |
| 6 (11.1%) cases | | | | | | + | + | – | – |
| 5 (9.3%) cases | | | | | | + | – | – | – |
| 2 (3.7%) cases | | | | | | – | + | – | – |
| 38 (70.4%) cases | | | | | | – | – | – | – |
| *34 cases with wild-type p53 did not have IHC* | | | | | | | | | |

[a]Accumulation of the wild-type/mutant form of p53 was detected by two monoclonal antibodies. The positive results were defined by consistently displaying positive signals using both antibodies. The results obtained by both antibodies demonstrated a very high degree of consistency, and only two cases yielded conflicting results. [b]Immunohistochemistry. ND, Not done.

specimens, which represented approximately 60% of the missense mutants. Further review of the database containing more than 300 p53 mutations in breast cancer (Cariello et al, 1994) showed that the pattern of change of codon/amino acid in three of our cases (cases 024, 068 and 089) have never been reported. We also identified three cases with frame-shift mutation due to deletion or insertion, and the sites of mutation in two of them, i.e. cases 12 and 97, have not been previously reported. Analysis of the prevalence and spectra of p53 mutations shows that nucleotide transition, including G:C → A:T (at either CpG or nonCpG sites) and A:T → G:C, is the major pattern found (Table 3), constituting 65% of total p53 mutations identified.

This study found the proportion of positive IHC (which indicates the presence of either wild-type or mutant p53 accumulation) to be 100% (14/14), 0% (0/4) and 29.7% (16/54) in the missense mutant cases, nonsense/frame-shift mutant cases and wild-type cases (including two cases with silent mutation) respectively (Table 2). These figures result in an estimation increase in p53 protein accumulation of 38.3% (100% × 16/114 + 0% × 5/114 + 29.7% × 93/114) in 114 breast cancer patients. We also used antibody specific for the mutant p53 form, and we further examined the site (i.e. nucleus or cytoplasm) of positive IHC in individual tumour cells (Figures 4 and 5). In 14 cases with p53 missense mutation, the common pattern (eight cases) exhibited a phenotype of positive nuclear staining detected by antibodies against both wild-type/mutant forms and mutant form p53 but with negative cytoplasmic staining detected by either antibody (Table 2). Four cases with nonsense/frameshift mutations showed negative staining in any location detected by antibodies against wild-type/mutant and mutant p53. Two cases harbouring silent mutation were confirmed by negative staining of mutant p53, but wild-type p53 was found in case 064. On the other hand, in 54 cases with

© Cancer Research Campaign 1997

**Figure 3** A frame-shift mutation due to an insertion of adenine at codon 175 (case 097)

**Figure 4** Sequential dissection of a case of ductal carcinoma in situ. (**A**) A standard haematoxylin- and eosin-stained section (40×). (**B**) Positive (brown) staining using antibody against mutant p53 (40×). (**C**) p53 localized mostly to cytoplasm (400×). (**D**), negative control (400×)

undetectable p53 mutation in exons 5–8, negative staining was expected in most of them (38/54), nevertheless p53 accumulation could be detected in 16 cases (Table 2). On the basis of IHC detection by antibody specific to mutant p53, we classified these 16 cases into two groups: those with positive mutant p53 (three cases), suggesting the presence of mutation, and those without mutant p53 (13 cases), suggesting the accumulation of wild-type p53. In 13 cases without mutant p53, 11 cases showed positive nuclear staining and eight cases displayed cytoplasmic p53 accumulation.

### DISCUSSION

Proportions of p53 mutation (22.8%) and of p53 accumulation (38.3%) comparable with those in high incidence areas were found in 114 Taiwanese breast cancers. However, novel patterns of p53 abnormalities, including unique sites or types of mutation, and overexpression of p53 protein, were detected in this study.

Current evidence of the structure–function relationship of p53 (Cho et al, 1994) suggests that some mutants of p53 alter their function and confer upon cells a growth advantage. Previous reviews have noted that major mutational hotspots in breast cancer occur in three amino acids (175, 248 and 273) in p53 (Greenblatt et al, 1994), the codons of which are all known to be related to specific functions of p53. The affected codons found in this study involved amino acids related to the role of p53 as a transcription factor binding specific DNA sequences (e.g. codon 248 in cases 077 and 092, codon 273 in cases 132 and 163) or to bind $Zn^{2+}$ (e.g. codon 179 in cases 089 and 115), and related to maintaining wild-type p53 conformation (e.g. codon 175 in cases 060 and 104, codon 249 in case 24). Subsequently, genomic alterations in these amino acids may result in a failure to transcribe cell-cycle regulatory proteins

**Table 3** Spectra of p53 mutation in exons 5–8 in breast cancer in Taiwan and other populations[a]

| | | Transitions | | | Transversions | | | |
|---|---|---|---|---|---|---|---|---|
| **Percentage of mutation (no. of tumours tested)** | **Deletion/ insertion** | **G:C→A:T at CpG** | **G:C→A:T at non-CpG** | **A:T→G:C** | **G:C→C:G** | **G:C→T:A** | **A:T→C:G** | **A:T→T:A** |
| Taiwanese 22.8 (26) | 11.5 (3) | 30.8 (8) | 19.2 (5) | 15.4 (4) | 7.7 (2) | 7.7 (2) | 3.9 (1) | 3.9 (1) |
| Current p53 mutation found 22.0 (338) | 16 | 23 | 13 | 11 | 8 | 13 | 6 | 7 |
| Caucasian in Europe 22.9 (122) | 11.5 | 23.7 | 18.2 | 8.8 | 7.4 | 18.2 | 8.1 | 4.1 |
| Caucasian in the United States 23.7 (60) | 11.0 | 19.2 | 23.3 | 23.3 | 11.0 | 5.5 | 4.1 | 2.7 |
| Japanese 20.1 (63) | 17.1 | 23.4 | 17.0 | 19.2 | 4.3 | 2.1 | 6.4 | 10.6 |

[a]The results of 21 studies conducted to examine p53 mutation in breast cancer published in 1990–95 have been included in this table. Incomplete information, e.g. mutation without confirmation by direct sequencing, is not included.

© Cancer Research Campaign 1997 

**Figure 5** Two cases of primary infiltrating ductal carcinoma stained for p53 using antibody against wild/mutant p53. (**A**) p53 shows largely nuclear localization. (**B**) p53 is localized largely within the cytoplasm

(e.g. p21) (el-Deiry, et al, 1994), and gain of oncogenic function. More specifically, experimental evidence indicates that at codon 175, only one of the two mutations (i.e. C:G → T:A and G:C → A:T transitions) obtain selective growth advantage (Arg → His but not Arg → Cys) (Greenblatt et al, 1994), which is consistent with our finding that, in cases 060 and 104, only Arg → His was observed. On the other hand, the consequence of other mutants with 'non-hotspot' missense mutation or intronic mutation must be evaluated at the p53 protein level.

Compared with p53 mutation spectra in other populations (Table 3), largely from high-incidence areas, our breast cancers with mutant p53 were characterized by a higher proportion of G:C → A:T transition at the CpG site, suggesting that different mechanisms are involved in our breast cancers. Given that G:C → A:T mutation occurs largely at sites where cytosine is methylated (i.e. the CpG site), experimental work and observation in humans strongly suggest that deamination of 5-methylcytosine (as opposed to mispairing of an $O^6$-methylguanine adduct with thymine) is the usual mechanism by which an G:C → A:T transition is found at a CpG dinucleotide in human tumour (Greenblatt et al, 1994). Spontaneous deamination of DNA (Wink et al, 1991), endogenous exposure to nitric oxide (Wink et al, 1991; Liu and Hotchkiss, 1995) and endogenous methylation driven by methyltransferase and *S*-adenosylmethionine (Shen et al, 1992) are three possible causes for deamination, and are currently under examination in our ongoing study.

The endogenous mechanism of slipped mispairing (Ripley 1990; Greenblatt et al, 1996) can be readily applied to explain frameshift mutation of single-base deletion and insertion in our cases, because the sites of mutation were located at 2-bp DNA motifs.

The accumulation of missense p53 mutant in cells is due to conformational changes in the mutant p53 polypeptide resulting in increased stability, whereas wild-type p53 protein or nonsense/ frameshift p53 mutants have a short half-life and are not usually detected by IHC. Therefore, most of the missense mutations, either at hotspots or non-hotspots, showed positive nuclear IHC, and all nonsense/frame-shift mutations show negative IHC. However, it has to be recognized that tumorous accumulation of p53 may reflect more the environment of the tumour cell than simply the intrinsic structure of the protein (Hall and Lane, 1994). The unexpected differences in IHC phenotypes in cases with same types of mutations may reflect this fact. Furthermore, an important issue concerning heterogeneity of tumour cells has to be raised. We found that not all staining was uniform and, even within a single microscopic field, some cells showed intensive positive staining whereas other cells were weakly positive or negative. This issue of heterogeneity can also be demonstrated by three of our cases with missense mutation (cases 69, 77 and 89), whose IHC phenotypes showed the possibility of coexistence of accumulation of both wild-type and mutant forms of p53.

It is interesting to note that a significant proportion (16/54) of our wild-type breast cancer revealed p53 protein expression in the nucleus or cytoplasm. These cases may harbour mutations in p53 exons not included in our analysis. However, an undetectable level of the mutant form of p53 by mutant-specific antibody indicates the other possibility that most of these proteins were wild-type p53. Further examination to locate this protein reveals a significant variation, and different locations may reflect different mechanisms. Normal lactating breast tissue has been shown to accumulate p53 in the cytoplasm of ductal cells, suggesting a distinct mechanism to inactivate p53 (Moll et al, 1992). Breast cancers that contain the wild-type form of p53 protein may inactivate its tumour-suppressing activity by stabilizing this protein in the nucleus or in the cytoplasm. The presence of p53-complexing proteins, such as MDM2 (Momand et al, 1992) or immediate–early 2 protein of human cytomegalovirus (a breast milk-transmitted virus) (Speir et al, 1994) in these tumours is currently under investigation.

The findings suggest that distinct mechanisms or different carcinogens may be involved in breast cancer in this low-incidence area. (The study of more cases is needed to confirm these results.

### ACKNOWLEDGEMENTS

We are greatful to Drs Yih-Horng Shiao and Jeou-Yoan Chen for valuable suggestions and criticisms during the course of this work.

### REFERENCES

Cariello NF, Cui L, Beroud C and Soussi T (1994) Database and software for the analysis of mutations in the human p53 gene. *Cancer Res*, **54**: 4454–4460 (This dataset has been updated by us, adding mutations published in 1995–96)

Cho Y, Gorina S, Jeffrey PD and Pavletich NP (1994) Crystal structure of a p53 tumor suppressor–DNA complex: understanding tumorigenic mutations. *Science* **265**: 346–355

El-Deiry WS, Harper JW, O'Connor PM, Velculescu VE, Canman CE, Jackman J, Pietenpol JA, Burrel M, Hill DE, Wang Y, Wiman KG, Mercer WW, Kastan

© Cancer Research Campaign 1997

MB, Kohn KW, Elledge SJ, Kinzler KW And Vogelstein B (1994) WAF1/CIP1 is induced in p53-mediated $G_1$ arrest and apoptosis. *Cancer Res* **54**: 1169–1174

Greenblatt MS, Bennett WP, Hollstein M and Harris CC (1994) Mutations in the p53 tumor suppressor gene: clues to cancer etiology and molecular pathogenesis. *Cancer Res* **54**: 4855–4878

Greenblatt MS, Grollman AP and Harris CC (1996). Deletions and insertions in the p53 tumor suppressor gene in human cancers: confirmation of the DNA polymerase slippage/ misalignment model. *Cancer Res* **56**: 2130–2136

Hall PA and Lane DP (1994) p53 in tumor pathology: can we trust immunohistochemistry: revisited. *J Pathol* **172**: 1–4

Liu RH and Hotchkiss JH (1995). Potential genotoxicity of chronically elevated nitric oxide: a review. *Mutation Res* **339**: 73–89

Moll UM, Riou G and Levine AJ (1992) Two distinct mechanisms alter p53 in breast cancer: mutation and nuclear exclusion. *Proc Natl Acad Sci USA* **89**: 7262–7266

Momand J, Zambetti GP, Olson DC, George D and Levine AJ (1992) The mdm-2 oncogene product forms a complex with the p53 protein and inhibits p53-mediated transactivation. *Cell* **69**: 1237–1245

Orita M, Iwahana H, Kanazawa H, Hayashi K and Sekiya T (1989) Detection of polymorphisms of human DNA by gel electrophoresis as single-strand conformation polymorphisms. *Proc Natl Acad Sci USA* **86**: 2766–2770

Pike MC, Spicer DV, Dahmoush L and Press MF (1993) Estrogens, progestogens, normal breast cell proliferation, and breast cancer risk. *Epidemiol Rev* **15**: 17–35

Ripley LS (1990). Frameshift mutation: determinants of specificity. *Annu Rev Genet* **24**: 189–213

Shen CY, HO MS, Chang SF, Yen MS, Ng HT, Huang ES and Wu CW. High rate of concurrent genital infections with human cytomegalovirus and human papillomaviruses in cervical cancer patients (1993) *J Infect Dis* **168**: 449–452

Shen JC, Rideout WM, III and Jones PA (1992) High frequency mutagenesis by a DNA methyltransferase. *Cell* **71**: 1073–1080

Speir E, Modali R, Huang ES, Leon MB, Shawl F, Finkel T and Epstein SE (1994) Potential role of human cytomegalovirus and p53 interaction in coronary restenosis. *Science* **265**: 391–394

Wink DA, Kasprzak KS, Maragos CM, Elespuru RK, Misra M, Dunams TM, Cebula TA, Koch WH, Andrews AW, Allen JS and Keefer LK (1991) DNA deaminating ability and genotoxicity of nitric oxide and its progenitors. *Science* **254**: 1001–1003.

Yang PS, Yang TL, Liu CL, Wu CW and Shen CY (1997) Risk factors for breast cancer in Taiwan-experience from a low-incidence area. *Br J Cancer* **75**: 752–756

© Cancer Research Campaign 1997